# Availability and readiness of primary healthcare facilities for the management of non-communicable diseases in different districts of Punjab, Pakistan

**DOI:** 10.3389/fpubh.2023.1037946

**Published:** 2023-03-10

**Authors:** Sadaf Rashid, Humaira Mahmood, Asma Asma Iftikhar, Nimrah komal, Zikria Butt, Hassan Mumtaz, Duha Shellah

**Affiliations:** ^1^Department of Public Health, National University of Medical Sciences (NUMS), Rawalpindi, Punjab, Pakistan; ^2^Department of Public Health, Health Services Academy, Islamabad, Islamabad, Pakistan; ^3^Department of Medicine & Health Sciences, An-Najah National University, Nablus, Palestine

**Keywords:** non-communicable disease, primary health care facility, SARA, public health, basic health units (BHUs)

## Abstract

**Introduction:**

Non-communicable diseases (NCDs) and their effects are rising quickly. NCDs such as cardiovascular illnesses, diabetes, cancer, and chronic lung diseases cause 60% of global deaths; of which, 80% occur in developing countries. In established health systems, primary healthcare handles most of the NCD care.

**Methodology:**

This is a mixed-method study conducted to analyze the health service availability and readiness toward NCDs using the SARA tool. It included 25 basic health units (BHUs) of Punjab, which were selected through random sampling. Quantitative data were collected using the SARA tools, while qualitative data were collected through in-depth interviews with healthcare providers working at the BHUs.

**Results:**

There was a problem of load shedding of both electricity and water in 52% of the BHUs, which leads to the poor availability of healthcare services. Only eight (32%) out of 25 BHUs provide the diagnosis or management of NCDs. The service availability was the highest for diabetes mellitus (72%), followed by cardiovascular disease (52%) and then chronic respiratory disease (40%). No services were available for cancer at the BHU level.

**Conclusion:**

This study raises issues and questions about the primary healthcare system in Punjab in two areas: first, the overall performance system, and second, the readiness of basic healthcare institutions to treat NCDs. The data show that there are many persisting primary healthcare (PHC) deficiencies. The study found a major training and resource deficit (guidelines and promotional materials). Therefore, it is important to include NCD prevention and control training in district training activities. NCDs are underrecognized in primary healthcare (PHC).

## Introduction

The incidence of non-communicable diseases (NCDs) as well as the damage they cause is increasing at a rapid pace. Recently, NCDs such as cardiovascular diseases, diabetes, cancer, and chronic lung diseases are responsible for approximately 60% of all deaths that occur across the globe, with 80% of these deaths taking place in developing counties (1). Primary healthcare (PHC) is vital to achieve UHC and decrease the prevalence of NCDs (2).

The majority of the care for NCDs is handled by primary care in highly developed health systems; however, the majority of primary care institutions in less-developed settings is not equipped to diagnose and treat NCDs. Only 6% of low-income nations compared with 85% of high-income countries have the requisite equipment to take six basic primary care assessments (3). Developing countries face an increase in NCDs from 1990 to 2010, with a 14% decrease in communicable diseases. As of now, NCD is the leading cause of death; therefore, health systems must be strengthened and prepared to tackle the disease burden of NCDs (4).

To reduce NCDs' impact on individuals and society, all sectors, including health, must work together. Low-cost options exist to minimize modifiable risk factors (primarily cigarette use, bad food, physical inactivity, and hazardous alcohol use) and map the NCD epidemic and risk variables. Primary care can provide high-impact NCD medicines for early detection and treatment. Creating healthy public policies and reorienting health systems can have the largest impact. The prevention and control of NCDs are less effective in low-income countries. High-income countries cover four times more NCD services than low-income countries. The WHO's Worldwide Action Plan for the Prevention and Control of NCDs 2013–2020 aims to reduce premature deaths from NCDs by 25% by 2025 through nine voluntary worldwide targets, including tobacco use, harmful alcohol use, unhealthy eating, and physical inactivity (5).

The WHO recommendations of essential medicines should be available to up to 80% of people as part of the global action plan for NCDs. The WHO has developed the service availability and readiness assessment (SARA) tool for collecting data on equipment, health workforce, and readiness and serves as an accountability and evaluative tool (6).

Our study aims to assess the availability of equipment, the workforce of primary healthcare services for their readiness toward the increasing burden of NCDs, and perceptions of healthcare providers toward the provision and upgrading of services.

## Methods

This study is a cross-sectional mixed method. Quantitative data were collected from 25 basic health units of different districts of Punjab. The sample size estimation was carried out after a thorough literature search using the SARA tool, and the estimate was based on a similar study conducted in Bangladesh (6). BHUs were chosen through the multistage sampling technique. In the first stage, eight districts were chosen by the lottery method, and in the second stage, three BHUs were randomly chosen (the lottery method) from sampled districts. An additional BHU was chosen for pilot testing of feasibility. The WHO's service availability and readiness assessment (SARA) tool was used for collecting quantitative data. The SARA tool general service readiness was measured by responses on the basic amenities and equipment: standard precautions for infection prevention, diagnostic capacity, availability of basic tests (hemoglobin, blood glucose, malaria diagnostic capacity, urine dipstick for protein, urine dipstick for glucose, HIV diagnostic capacity, syphilis RDT, and urine pregnancy test), and availability of essential medicines. Service-specific readiness for NCDs was measured through the availability of diagnostic and management services, pieces of training, and guidelines for the treatment of common NCDs (diabetes, cardiovascular disease, chronic respiratory disease, and cervical cancer).

A pilot study was conducted to look into the feasibility of data collection. The participants took almost 10–15 min to complete the informed consent and questionnaire. An incomplete questionnaire was excluded from the final analysis.

For collecting qualitative data, one focus group discussion (FGD) () with 10 medical officers currently working in BHUs and one in-depth interview with MS were conducted. Purposive sampling was used. Detailed notes were taken, and interviews were recorded with the permission of the participants. An interview guide was used to probe problems regarding the provision of services, suggestions for improvement, and public–private partnerships. Data were collected till the saturation of response. It was later transcribed, and themes and subthemes were identified through the coding of the response.

## Results

### Demographic description

In this study, 25 BHUs from the rural setups were included to analyze the readiness of primary healthcare facilities for the management of NCDs in different districts of Punjab. Two BHUs (8%) were taken from each Basti Alamgir, Basti Malook, Kotla Faqir, Bhatia, and Darapur districts. Three BHUs (12%) were taken from each Jandala, Multan, and Sagri districts. One BHU (4%) was taken from each Jalalabad, Khairabad, and Mubarikpur.

### Service availability at BHUs of Punjab district

General health infrastructure and health service providers at BHUs. Service availability was analyzed by accessing the infrastructure and health workforce available at the BHUs included in the study. The availability of overnight inpatient beds was not present in 19 (76%) BHUs, while one (4%) BHUs have one inpatient bed availability, and five (20%) BHUs have three inpatient overnight beds. Almost all the BHUs available in our study had dedicated maternity beds. Ten BHUs (40%) have one dedicated maternity bed, and 15 BHUs have two dedicated maternity beds.

All the BHUs included in our study have only one general practitioner each, and none of the BHUs have any medical specialist, pharmacist, or lab technologist. Almost 15 (60%) BHUs do not have any nursing facilities. Five (20%) BHUs have only one nurse available, and five (20%) BHUs have two nurses available at their unit. Midwives were present in all the BHUs, five (20%) BHUs have two midwives, five (20%) BHUs have three midwives, five (20%) BHUs have four midwives, and 10 (40%) BHUs have five midwives each. Community health workers were present in all BHUs included in our study.

### Service readiness

#### Communication and transport

Service readiness was analyzed by accessing the availability of modes of communication, access to ambulances in need of emergency situations, power supply and water supply resources, rooms with privacy, and adequate sanitation amenities. Ten (40%) BHUs have 24 h available functioning landline facility, whereas 15 (60%) BHUs do not have this facility. Twelve BHUs (48%) have functioning mobile phones dedicated to the facility, while 13 (52%) BHUs do not have this facility. None of the BHUs have functioning short-wave radio for radio calls.

A total of 11 (44%) BHUs have functioning computers available in the facility, while 14 (56%) BHUs lack this facility. Notably, 19 (76%) BHUs have email access or Internet within the facility, while six (24%) BHUs did not have any Internet access. In regard to ambulance access, 10 (40%) BHUs have a functioning ambulance for the transport of patients stationed at the facility, while 15 (60%) BHUs do not have this facility. In total, 17(68%) BHUs have the access to ambulances/emergency vehicles stationed at nearby facilities, while eight (32%) BHUs did not have such facilities. In regard to fuel for the ambulance, 10 (40%) BHUs have the fuel for the ambulance/emergency vehicles available on the day of the interview, while 15 (60%) BHUs do not have the fuel facility.

#### Power supply, water supply, sanitation, and privacy

The power supply of all the BHUs was analyzed by checking the sources of electricity at the BHUs and their hours of availability. It was observed that all the BHUs were having a central electrical supply from the national grid. Seven (28%) BHUs were using only standalone electric medical devices/appliances (e.g., epi cold room, refrigerator, and suction apparatus), while the remaining 18 (72%) were fulfilling all the electrical needs of the facility from the main supply. A total of 12(48%) BHUs do not have the facility of a functional generator if the main source of supply is not functional, and the remaining 13 (52%) do not know whether the unit has a functional generator or not. Three out of the 25 BHUs included in the study have a generator (fuel or battery-operated generator) as a secondary or backup source of electricity, while 18 have a solar system as a backup (72%). Electricity is always available in 12 BHUs (48%), while it is frequently available in 13 (52%) BHUs. In regard to the water supply, 20 (80%) BHUs have a 5- to 8-h water supply, and five (20%) BHUs have a 24-h water supply. Five (20%) BHUs used supply water as the most commonly used source of water for the facility at the time of the interview, and 25 BHUs used tube well or borehole water as the most commonly used source of water for the facility at the time of the interview. Seven (28%) BHUs have auditory privacy, and 18 (72%) BHUs have both auditory and visual privacies. All of the 25 BHUs have a flush toilet (latrine) within the premises in functioning condition and accessible for general outpatient client use.

#### Reuse of equipment and healthcare waste management

Processing equipment for reuse is an important parameter for the good hygiene of a health unit. We analyzed the presence of some processing equipment in the 25 BHUs included in our study. Four (16%) BHUs do not have any electric autoclave, while three (72%) have a non-functioning electric autoclave, and 18 (72%) have a properly functioning electric autoclave. In total, 17(68%) out of 25 BHUs do not have the facility of a non-electric autoclave, while three (12%) have a non-functioning non-electric autoclave, and five (20%) BHUs have a fully functional non-electric autoclave. Seven (28%) BHUs do not have an electric dry heat sterilizer, while three (72%) have a non-functioning electric dry heat sterilizer, and 15 (60%) have a properly functioning electric dry heat sterilizer.

Five (20%) out of 25 BHUs do not have the facility of electric boilers or steamers, while 20 (80%) BHUs have fully functional electric boilers or steamers. Non-electric pots with covers for boiling and steaming were not available in 17 (68%) BHUs, while eight (32%) have a fully functional non-electric pot. The heat source for non-electric equipment was not available in 24 (96%) BHUs, while only one BHU has its availability. Of note, 11 BHUs used two-chamber burn incinerators for sharp waste disposal, 10 BHUs used open burning in a pit or protected ground, and four BHUs used dump without burning in a covered pit.

### Basic equipment

The readiness of basic equipment is a very important parameter in accessing the quality of primary healthcare. Out of the 25 BHUs, 22 (88%) have a properly functioning adult weighing scale, while only three (12%) BHUs do not have this basic equipment. A total of 17 (68%) BHUs have child weighing scales, while eight (32%) do not have this facility. Notably, 18(72%) BHUs have the infant weighing scale, while seven (28%) do not have this basic equipment. Stadiometer was available at 20 (80%) BHUs and not available at five (20%) BHUs. A thermometer, stethoscope, and blood pressure apparatus were present in all the included BHUs. Nine (36%) BHUs do not have the facility of oxygen concentrators, while 16 (64%) BHUs were equipped with this device. All of the 25 BHUs included in the study were having oxygen cylinders. The central oxygen supply was not available in 17 (68%) BHUs, while eight (32%) BHUs were equipped with this facility. In total, 12 (48%) out of the 25 BHUs have the flowmeter with oxygen, while 13 (52%) BHUs were deprived of it. Oxygen delivery apparatus was available in 22 (88%) BHUs and absent in three (12%) BHUs. Intravenous infusion kits were functioning properly in 20 (80%) BHUs, while it was not available in five (20%) BHUs.

#### Laboratory diagnostic facility

Laboratory testing is one of the principal tools in the diagnosis of a particular disease. The availability of these testing facilities at the level of BHUs is one of the important parameters in a primary healthcare management system. In our study, 17 (68%) out of the 25 included BHUs, and an onsite diagnostic testing facility was available. The facility for blood glucose tests using a glucometer was available at the BHUs. A total of 12 (48%) BHUs had urine glucose dipstick testing and hemoglobin testing was taking place at 22 (88%) BHUs. General microscopy was taking place at six (24%) BHUs. Stool routine examination was taking place at eight (32%) BHUs. ABO blood grouping, rhesus blood grouping, urine microscopy testing, and blood group serology testing facilities were available at seven (28%) BHUs.

None of the BHUs were providing urine protein dipstick testing, urine ketone dipstick testing, special renal function test, liver function tests cross matching, serum electrolyte testing, and CSF/body fluid testing. The availability of general and special laboratory equipment along with laboratory reagents was analyzed. It was reported that six out of 25 BHUs have a fully functional light microscope. The availability of glass slides and cover slips was 20% (five out of 25 BHUs). Out of 25 BHUs, seven (28%) BHUs have a fully functional refrigerator, 13 (52%) BHUs have a non-functioning refrigerator, and five (20%) BHUs do not have a refrigerator facility.

Seven (28%) BHUs have the facility of a glucometer, five (20%) BHUs do not have the facility of the glucometer, and 13 (52%) BHUs have a non-functioning glucometer. Glucometer test strips were available at seven (28%) BHUs, while in 13 (52%) BHUs non functioning glucometer test strips were available, and five (20%) BHUs lacked this facility. Colorimeter or hemoglobinometer was available at four (16%) BHUs, five (20%) BHUs have non-functioning colorimeters, while 16 (64%) do not have the facility of a colorimeter.

The incubator was available in a non-functioning condition in nine (36%) BHUs, and 16 (64%) BHUs do not have the facility. The centrifuge was available in a functioning condition at five (20%) BHUs. White blood counting chamber was present in a functioning condition at six (24%) BHUs.

#### Imaging diagnostic facility

The availability of imaging diagnostic facilities was analyzed in all the BHUs included in this study. Out of 25 BHUs, only six (24%) have the facility of imaging diagnostics. None of the BHUs were performing CT scans or MRIs as they do not have the equipment. A total of 19 (76%) out of 25 BHUs do not have the facility of x-rays and ultrasound imaging. Two (8%) have x-rays and ultrasound machines but they are not in a functioning condition, while four (16%) BHUs have the proper functioning x-rays and ultrasound machine setups. Two (8%) BHUs have the facility of a properly functioning ECG machine, while three (12%) BHUs have a non-functional ECG machine.

#### Indicators for non-communicable disease service availability and readiness

Service availability and service readiness at the primary healthcare levels or at the level of basic health units for the treatment, management, and prevention of NCDs: NCDs are emerging at a greater pace in Pakistan. The included 25 BHUs in the current study were accessed to check how many services these BHUs provide against NCDs. The availability of treatment and management of diabetes mellitus, cardiovascular disease, and respiratory disease was assessed. Eight (32%) out of 25 BHUs provide the diagnosis or management of NCDs.

### Diabetes mellitus

Diagnosis and management of diabetes mellitus in patients were available at 18 (72%) BHUs. A total of 15(60%) BHUs have national guidelines for the diagnosis and management of diabetes mellitus. Eight (32%) BHUs have attempted the diagnosis and management of diabetes mellitus in the last 2 years. Service readiness for the treatment and management of diabetes mellitus was analyzed by accessing the equipment, diagnostic facilities, and medicines that are required for the management and treatment of diabetes mellitus. It was reported that all BHUs have the basic equipment including a stethoscope and the blood pressure apparatus, while 22 (88%) BHUs have the facility of an adult weighing scale, and 20 (80%) BHUs have a fully functional stadiometer. Onsite testing of blood glucose was present in all the BHUs. A urine glucose dipstick test was available at 12 (48%) BHUs.

Medicines and commodities related to diabetes mellitus include gliclazide tablet or glipizide tablet (not available at any of the BHUs), metformin (available at all BHUs), insulin regular (available at seven (28%) BHUs), glucose 50% injection (available at six (24%) BHUs), and glibenclamide cap/tab (available at 12 (48%) BHUs).

### Cardiovascular diseases

In total, 13(52%) BHUs have the facility to diagnose and manage cardiovascular diseases such as hypertension in patients. Three (12%) BHUs have attempted the diagnosis and management of cardiovascular diseases in the last 2 years. Eight (32%) have the national guidelines for cardiovascular disease diagnosis and management. Service readiness against the treatment and management of cardiovascular disease was analyzed by accessing the equipment, diagnostic facilities, and medicines that are required for the management and treatment of diabetes mellitus. It was reported that all BHUs have the basic equipment including a stethoscope, the blood pressure apparatus, and oxygen cylinders, while only 22 (88%) BHUs have the facility of an adult weighing scale. A properly functioning ECG machine was available at only two (8%) BHUs. Medicines and commodities related to cardiovascular disease include metformin, aspirin, and beta-blockers (available at all BHUs), and ACE inhibitors and calcium channel blockers were not available at any of the BHUs.

### Chronic respiratory diseases

A total of 10 (40%) BHUs diagnose and manage chronic respiratory diseases in patients. Eight (32%) BHUs have national guidelines for chronic respiratory disease diagnosis and management. Three (12%) BHUs have attempted diagnosis and management of CRD in the last 2 years. Peak flow meters were present in seven (28%) BHUs. Service readiness against the treatment and management of cardiovascular disease was analyzed by accessing the equipment, diagnostic facilities, and medicines that are required for the management and treatment of chronic respiratory disease. Peak flow meters were present in seven (28%) BHUs. Stethoscopes, oxygen cylinders, and blood pressure apparatuses were present in all the BHUs. Oxygen concentrators were present in 16 (64%) BHUs.

Medicines and commodities for chronic respiratory diseases include prednisolone and epinephrine injection (both available at six (24%) BHUs), and hydrocortisone injections were present in all the BHUs. None of the BHUs have salbutamol and beclomethasone inhaler.

### Cervical cancer

None of the BHUs provide the facility to diagnose cervical cancer in patients. These BHUs neither have the national guidelines for cervical cancer prevention and control nor training in cervical cancer prevention and control. The availability of auto-disable syringes was present in 15 (60%) BHUs.

Two focal group discussions having (8–12) participants including the general practitioners, nurses, medical officers, and community health workers vaccinators were done to qualitatively analyze the primary healthcare services and availability at the BHUs of Punjab district for the NCDs. The collected data were analyzed through thematic analysis.

### Health service availability for the treatment and management of NCDs at BHUs

Available guidelines for the treatment and management of NCDs include diabetes mellitus, cardiovascular disease, chronic respiratory disease, and cancer: Regarding the service, only the practitioner were available for the management and treatment of NCDs at primary health levels that includes the BHUs, a detailed interview was conducted with the group of the service provider at the BHUs that infer that almost all the BHUs included in our study do not have the availability of medical specials, general doctors, and practitioners were only there. The management and treatment of NCDs were only offered at 24% of the BHUs, and the reasons that the staff shared were the lack of specialized staff at the BHUs that include medical specialists, pharmacists, and medical technologists. The staff further adds that there is less accountability of the staff at the BHU level.

During the interview, it was also reported that treatment and management services were available at maximum BHUs, but they still lack the availability of a diabetic specialist.

They said that “*BHUs lack professionals. It's hard to find doctors with NCD expertise to operate in community health stations.”*

They further added that “*The budget for NCD primary healthcare is quite limited; funding is mostly from national goal programs, but they've been slashed.”*

Services related to cardiovascular diseases were not present in half of the BHUs due to the non-availability of specialists and lack of staff. The interview also reported that national guidelines for NCDs were not present in all the BHUs. A smaller number of BHUs have received such guidelines, that is, the main reason quoted by the staff and healthcare workers at the BHUs was the lack of interest of the government officials toward the BHUs and the lack of awareness regarding the NCDs. Even a small number of the staff had an awareness of the national strategies regarding the guidelines against the NCDs.

### Problems regarding the service available at the BHUs

The main reported problem during the in-depth interview appeared to be the lack of proper infrastructure of the BHUs, either general or medical. Maximum BHUs lack the trained staff for the NCDs. This results in weak monitoring of the NCDs at the primary healthcare level (BHUs).

### Suggestions for improvement

The provision of a good infrastructure including a good building with proper ventilation and sanitation along with an efficient group of specialists including a medical specialist, a lab technologist, and a pharmacist along with the midwives and community health workers can improve the level of service available at the level of BHUs.

### Service readiness in terms of power supply, water supply, communication, and transport

Problems regarding power supply, water supply, communication, and transport: The main concern of the health service providers at the BHUs was the availability of landline connections for better communication among the public and healthcare providers. Many of the BHUs do not even have an ambulance facility. The workers also shared the problems of load shedding of electricity at maximum BHUs and also shared their problems that their routine is very much disturbed at the BHUs as they are unable to perform their duties without electricity as many BHUs do not have an electricity backup, for example, a generator. During the interview, water shortage was also reported at maximum BHUs.

### Suggestions for improvement

It was suggested during the interview that service readiness can be improved for the public if there is an availability of an ambulance at all the BHUs and if there is the provision of a backup supply for electricity in case of load shedding. For a better mode of communication, they suggested a 24 h availability of the landline connection.

### Service readiness in terms of imaging, laboratory diagnostics, and medicines for NCDs

Problems regarding imaging, laboratory diagnostics, and medicines at BHUs: Regarding imaging diagnostic services, maximum BHUs do not have the facility to provision of imaging and laboratory diagnostics. Moreover, those BHUs that have this facility have non-functional x-ray and ultrasound machines. Onsite blood testing was available at the BHUs, but not all the basic tests are performed at all the BHUs as they lack the equipment for special tests.

They said that,

“*Our BHU setup do[sic] not stock any medications. What we do have are medications designed for use in emergencies.”*

In regard to medicine some BHUs lacked the basic medicines for the NCDs, the reasons shared during the interview for these problems were mainly the negligence of the government official toward BHUs.

#### Suggestions for improvement

Suggestions gathered during the interviews for the problems related to laboratory and imaging diagnostics were mainly the repair of the non-functioning equipment and machines used for imaging and laboratory testing. The BHUs that do not have the imaging and the laboratory facility suggested induction of these machineries at the BHUs. The staff also suggested a good supply of medicine for the NCDs so that they are easily accessible to the public.

## Discussion

The study explored the service readiness of primary healthcare facilities for the management of NCDs in different districts of Punjab. Our study showed that non-communicable diseases are a burden on the economy and healthcare system of Pakistan. The current study analyzed the readiness of the basic health units toward the NCDs.

Non-communicable diseases are a burden on the economy and healthcare system of Pakistan. The current study analyzed the readiness of the basic health units toward the NCDs. It just had a general practitioner, nurse, midwives, and some community health workers. Researchers in Bangladesh, Haiti, Malawi, Nepal, and Tanzania found that a few institutions were entirely “equipped” to perform any one NCD service (7).

The purpose of this study was to assess the existing readiness of facilities in basic health units to provide care for NCDs. The evaluation focused on analyzing two aspects of the NCD-specific services: the availability of the services and the readiness of the services for patients with NCDs. The service availability was the highest for diabetes mellitus (72%), followed by cardiovascular disease (52%) and chronic respiratory disease (40%). No services were available for cancer at the BHU level. The good prevalence score for CVD was the greatest (22.6%), followed by the readiness score for diabetes (17.2%). Other NCDs in the current study include diabetes, cardiovascular diseases, chronic respiratory diseases, and cancer (8).

Onsite testing of blood glucose was present in all the BHUs. Glibenclamide cap/tab was 19–52% available. A study found limited insulin availability (9–16% depending on insulin type), whereas another study found 34%. Captopril availability ranged from 13 to 48%, calcium channel blockers from 29 to 57%, and beta-blockers from 15 to 50% (9). The study in India found that the availability of all essential technology and medications in primary care ranged from 1.1% in rural public facilities to 9.0% in urban private facilities for the management of three NCDs. At present, neither private nor public primary care facilities, nor public secondary care facilities are fully prepared to effectively manage the burden of NCDs in India (10).

According to Nhsrc.pk 2019 report (11), 43% of doctors and 98% of support staff were present at the facilities (BHUs in Islamabad). Only 21% of facilities had blood sugar testing. Metformin, sulphonylureas, and insulin were not present in 65, 79, and 93% of the facilities. Thiazides and statins were not accessible anywhere, and beta-blockers were available in 29%. A total of 36% of the institutions possessed a computer, but only one utilized it. Only 14% of buildings had Internet. In rural areas, a model of reform at one BHU provides a framework for consolidation.

Concerns about the availability of NCD pharmaceuticals have been raised as a result of the findings of a second study, particularly in the public sector and rural areas. This has led to an inadequate supply of relatively affordable NCD medications. Metformin, glibenclamide, and other ACE inhibitors are no longer protected by patents and are available from multiple sources. In low- and middle-income countries, there is a concern regarding the availability of insulin and the price of insulin, with syringes and other diabetes goods adding to the burden of treatment (12).

## Limitations

We had some limitations while conducting the study. The first limitation is based on a small sample because of limited resources. The second limitation is based on the population. This study population may not be representative of all BHUs in Pakistan as it is covering only one province. Finally, the third limitation is based on the non-availability of the staff on the day of the interview at some BHUs.

## Conclusion

Improving efforts to prevent and control NCDs through BHUs (primary healthcare) in Punjab requires increased political commitment and financial investment at all levels of the health system. A major gap also exists in the primary healthcare (PHC) setting for the identification and treatment of NCDs. These findings indicate a sizable need that has to be filled, especially in terms of the provision of education and accessibility of tools.

## Recommendations and future research

A reorientation of the national health system is necessary to address the NCD burden and enhance NCD services at the primary healthcare center level. Workflow adjustments, duty distribution among primary care teams, recording patient information, and including community health workers in patient follow-up are all issues that plague primary care settings in India. The team's hierarchy inhibited quality improvements and team-based treatments. More studies on organizational behavior in primary care facilities in India are needed to help advance the state of primary healthcare in the country.

## Data availability statement

The raw data supporting the conclusions of this article will be made available by the authors, without undue reservation.

## Ethics statement

The studies involving human participants were reviewed and approved by National University of Medical Sciences. The patients/participants provided their written informed consent to participate in this study.

## Author contributions

All authors listed have made a substantial, direct, and intellectual contribution to the work and approved it for publication.
